# Supplementary material for: Complexome profiling of the Chlamydomonas psb28 mutant reveals TEF5 as an early PSII assembly factor
Source: Plant Cell. 2025 Mar 17;37(6):koaf055. doi: 10.1093/plcell/koaf055 (PMC12164590; doi:10.1093/plcell/koaf055)
Supplement: koaf055_Supplementary_Data [file koaf055_supplementary_data.zip › psb28_interactive.zip!/plots/intensity/qualityPlots/peptides/PMA2_Cre10.g459200.t1.2.html]

### Cre10.g459200.t1.2

Trivial name: PMA2  
SeqCoverage [median]: 16.30  
Mass: 117.28 kDa  
  
 

| Sequence | MissedCleavage | Mass [Da] | wtIntensity | muIntensity | Score |
| --- | --- | --- | --- | --- | --- |
| AAALISGTHNVANLQIIMTK | 0 | 2065.14 | 0.00 | 1212.62 | 79.68 |
| AADVGVTGMTVPTASNPLGR | 0 | 1912.97 | 0.00 | 898.20 | 55.44 |
| ADVGIAVAGATDAAR | 0 | 1356.70 | 762.02 | 731.41 | 102.40 |
| ALAGALAPK | 0 | 810.50 | 0.00 | 1001.57 | 97.07 |
| AYNKDTLADNVNAK | 1 | 1535.76 | 935.46 | 3538.55 | 130.47 |
| EALSILNTTPHGLSSAEADKR | 1 | 2209.13 | 0.00 | 1547.45 | 36.13 |
| FGDIVAADVK | 0 | 1033.54 | 0.00 | 4290.36 | 103.55 |
| GAADIVLTEAGLSAIK | 0 | 1527.85 | 413.69 | 0.00 | 77.60 |
| KADVGIAVAGATDAAR | 1 | 1484.79 | 0.00 | 780.07 | 71.35 |
| LAEYGPNRLPESK | 1 | 1472.76 | 0.00 | 698.10 | 62.14 |
| LNEGMQR | 0 | 846.40 | 0.00 | 246.97 | 98.42 |
| MLGMGTVMYPSEVLIK | 0 | 1767.90 | 0.00 | 1986.47 | 103.75 |
| MVEFANR | 0 | 865.41 | 0.00 | 1192.56 | 144.29 |
| TSDGLVR | 0 | 746.39 | 444.36 | 1378.70 | 83.61 |
|  |  |  | 2555.5 | 19503.0 |  |
